# Network Analysis of the Gender Gap in International Remittances by Migrants

**DOI:** 10.1007/s12626-022-00125-9

**Published:** 2022-10-01

**Authors:** Zelda Marquardt, Yuichi Ikeda

**Affiliations:** Graduate School of Advanced Integrated Studies in Human Survivability, Kyoto University, 606-8303 1 Nakaadachi-cho, Yoshida, Sakyo-ku Kyoto, Japan

**Keywords:** Financial inclusion, Gender gap, International Migration, International Remittances, Complex network, SVD, Bow-tie structure, Community detection

## Abstract

Financial inclusion is considered a key enabler of international development goals. Despite the expansion of financial access overall, the gender inequalities in basic access have remained consistent. This research investigates the predictive power of global remittance and migration flows on the gender gap in financial inclusion. First, singular value decomposition is applied to the World Bank’s 2017 Global Findex data to identify the financial inclusion variables that most contribute to the gender gap in financial inclusion. We find that indicators pertaining to account ownership, emergency funding, and receiving payments are especially significant. Based on the identified variables, a novel Financial Inclusion Gender Gap Score is calculated for 143 economies. The score is then incorporated into a complex network analysis of global remittance and migration networks. We analyze how network features such as node attributes, community membership, and bow-tie structure can be used to make inferences about the magnitude of a financial inclusion gender gap. Our findings suggest that weaker linkages in the network, characterized by lower node strength and peripheral positions in the bow-tie structure, are determinants of a notable financial inclusion gender gap. We also highlight communities in the remittance and migration networks with a more substantial gender imbalance, and discuss the the social- and cultural-leaning factors driving community formation in the migration network that seem to predicate a greater gap.

## Introduction

The importance of financial inclusion in the realization of international development goals has been well-recognized by researchers and policymakers alike. Financial inclusion refers to equitable and sustainable access to financial products and services. According to the most recent 2017 World Bank’s Global Findex Report, 1.7 billion people remain unbanked, meaning they hold no account with a financial institution or a mobile money provider. Even more people would be categorized as underbanked, meaning they have insufficient access to mainstream financial services such as insurance or loans. Despite the expansion of overall financial access in the past decade, gender inequalities in basic access (account ownership) have remained consistent at 7 percent globally and 9 percent for developing countries since 2011 [1]. This implies that there exist barriers specific to women that need to be addressed to achieve universal financial inclusion.

On the other hand, the rise in international migration and improvements in financial technology has greatly increased the quantity and value of international migrant remittances—money sent back to families and friends in their countries of origin. Remittance flows to low- and middle-income countries (LMICs) reached a record high of $548 billion in 2019, up from $529 billion in 2018 (+ 3.6%) [2]. Although this rise was reversed by the COVID-19 pandemic and the ensuing economic crisis, the importance of remittances as a source of external financing for LMICs is expected to continue its rise in coming years [3]. This has generated attention on the possibilities of migrant remittances as a catalyst for economic and social development.

While past research has investigated measures and determinants of financial inclusion at the local or country level, studies of macrolevel data across countries are still lacking. For research focused on gender and or remittances, the studies tend to be based on survey questionnaires or interviews conducted, often within a single neighborhood or district [4]. Country-level studies are crucial in assessing the local context for financial inclusion and capturing microlevel determinants. However, given that the growing influence of migrant remittances is a consequence of globalization—the increasing complexity and interconnectedness of heterogeneous systems—this approach is limited in its scope. Comparing performance across general regional classifications would likewise be limiting, as many of the largest migrant corridors are between countries in different geographic regions. Research that examines the determinants of the gender gap based on the systematic study of complex linkages between countries is therefore warranted.

For this reason, this study integrates network analysis—the study of complex patterns of connection—of global migrant remittance flows into its analysis of the gender gap in financial inclusion. Several studies on the dynamics of global remittance networks have been conducted. Lillo et al. [5] conducted a graph-based analysis of bilateral remittance data between 2010 and 2013; Ignacio & Darcy [6] studied Asian remittance and migration flow networks using 2015 data; and Wen et al. [7] did a complex network analysis of global remittances for the period 2010–2016. A close comparative study of global remittance and migration networks has yet to be conducted. Moreover, past studies discussed key features of the network but stopped short of addressing social issues or incorporating further datasets. Network science has not yet been used to dig deeper into financial inclusion solutions. This research therefore proposes a new understanding of financial inclusion as a function of their migrant and remittance flows.

This study also builds on literature around the use of statistical techniques to investigate the gender gap in financial inclusion. Several past studies have presented indices of financial inclusion, to explore ways of defining and calculating financial inclusion. However, traditional models have tended to limit variables to indicators like account ownership or number of ATMs available, and have not taken advantage of the wealth of microdata that is now available. Of these, several studies have applied statistical dimensionality reduction techniques to measure the most relevant variables [4, 8, 9]. The topic of gender inequality was incorporated by Aslan et al. [10] and Delechat et al. [4], who developed indices based select variables from the 2011 and 2014 waves of the Global Findex data. This study will add to this field by constructing its own novel global financial inclusion gender gap index based on the most recent data, incorporating all indicators available in the 2017 wave.

This study aims to empirically assess the global gender gap in financial inclusion through the lens of migrant remittance networks. This study will clarify (1) what financial inclusion indicators most contribute to the gender gap, (2) what features of remittance and migration networks predict gender gap performance at a regional level, and (3) what are determinants of gender gap performance at a country level. We first propose an original index for measuring the gender gap, developed based on results from Singular Value Decomposition (SVD) of the World Bank’s 2017 Global Findex data. The underlying reasons for the gender gap are revealed, and 143 economies are given a score—henceforth called the Financial Inclusion Gender Gap Score (FIGGS)—based on their contribution. To shed light on the international flow of migrants and their money, a global remittance network (GRN) and global migration network (GMN) based on 2017 World Bank data are constructed, analyzed, and compared. Finally, the FIGGS results are incorporated into the remittance and migration network to evaluate the predictive power of network attributes on the financial inclusion gender gap.

The remainder of this paper is organized as follows. Section 2 explains the data and methodology used. Section 3 describes the empirical results. Section 4 presents a discussion of the findings. Finally, Sect. 5 concludes.

## Data and Methodology

### Data

For financial inclusion data, the World Bank’s Global Findex Database is the world’s most comprehensive demand-side dataset measuring nearly 800 indicators through nationally representative surveys of more than 150,000 adults (men, women, and youths) in over 140 economies. In accordance with the World Bank’s classification, we use the term *economy* interchangeably with *country* to refer to any territory reporting separate economic statistics. The 2017 wave includes 45 indicators whose respondents can be gender-disaggregated, and provides data on how men and women save, borrow, make payments, and manage risk. For migrant remittances, the World Bank’s Bilateral Migration Matrix (2010, 2013, 2017) contains estimates of the total number of international migrants, disaggregated by migrant source economies, for 214 countries and territories [2]. The Bilateral Remittance Matrix contains annual bilateral migrant remittance estimates for the same 214 economies for 2010–2017.

### Financial Inclusion Gender Gap Score

#### Singular Value Decomposition

Singular Value Decomposition (SVD) is a technique often used for dimensionality reduction. SVD allows the transformation of a high-dimensional matrix into a low-dimensional one by eliminating the less important properties and retaining only the more important parts of a dataset [11]. An approximated representation is then constructed with any chosen number of dimensions.

SVD involves the factorization of any rectangular $m \times n$ matrix ${\mathbf {A}}$ as:

1 $$\begin{aligned} {\mathbf {A}}={\mathbf {U}} \mathbf {\Sigma } {\mathbf {V}} ^T, \end{aligned}$$

where ${\mathbf {U}}$ is orthogonal matrix $m \times m$, ${\mathbf {V}}$ is orthogonal matrix $n \times n$, and ${\mathbf {V}}^T$ is the transpose of matrix ${\mathbf {V}}$, found by exchanging the rows and columns of the matrix $\mathbf {\Sigma }$ is diagonal matrix $m \times n$, whose non-zero elements are called singular values of ${\mathbf {A}}$. The singular values of $\mathbf {\Sigma }$ are the square roots of eigenvalues of the $n \times m$ matrix, organized in decreasing order.

The number of singular values used to reconstruct the low-dimensional matrix determines the accuracy of the approximation, with fewer dimensions meaning a less accurate approximation. It is considered best to retain enough singular values to explain 90% of the variance of a dataset. By keeping the top $r$ singular values (and the top $r$ vectors in ${\mathbf {U}}$ and ${\mathbf {V}}$), an approximate matrix ${\mathbf {A}}_r$ can be constructed where orthogonal matrix ${\mathbf {U}}_r$ is $m \times r$, ${\mathbf {V}}_r$ is $r \times n$, and diagonal matrix $\mathbf {\Sigma }_r$ is $r \times r$. We can write this as:

2 $$\begin{aligned} {\mathbf {A}}_r={\mathbf {U}}_r \mathbf {\Sigma }_r {\mathbf {V}}_r ^T = {\mathbf {X}}_r {\mathbf {V}}_r ^T, \end{aligned}$$

where

3 $$\begin{aligned} {\mathbf {X}}_r={\mathbf {U}}_r \mathbf {\Sigma }_r. \end{aligned}$$

${\mathbf {X}}_r$ is, therefore, the orthogonal projection of ${\mathbf {A}}$ on ${\mathbf {V}}_r$ for the first r singular values. The higher values of each dimension in ${\mathbf {X}}_r$ helps expose the most useful and interesting properties of the dataset.

#### Extract Gender Gap

In the 2017 Global Findex dataset, 45 indicators can be gender-disaggregated. A full list of these indicators is available in Appendix I. To measure the indicators that most contribute to a gender gap, we compare $X_r$ for the “male” disaggregated set and the “female” disaggregated set. For each financial inclusion indicator $q$, we calculate the difference $D$ in male respondents-only indicators $q_m$ and female respondents-only indicators $q_f$ for each dimension $r$. The difference is adjusted by multiplying the difference expressed as eigenvalues by the difference expressed as percentage:

4 $$\begin{aligned} D_q^r = ({X_{q_f}^r}-{X_{q_m}^r})\frac{({X_{q_f}^r}-{X_{q_m}^r})}{X_{q_f}^r}. \end{aligned}$$

#### Extract Financial Inclusion Indicators

To adjust for the difference in ranges across each dimension of $D_q^r$ , the Z score is taken:

5 $$\begin{aligned} Z_q^r = \frac{D_q^r-\mu ^r}{\sigma ^r}, \end{aligned}$$

where $Z_q^r, D_q^r, \mu ^r , \sigma ^r$ are standard score, observed value, mean of sample, and standard deviation of sample, respectively. A threshold of $|Z_q^r|=1$ is set, and questions with Z scores above the threshold are identified as the most important indicators across all dimensions.

#### Assigning a Financial Inclusion Gender Gap Score

A Financial Inclusion Gender Gap Score “FIGGS” is assigned for each economy $V$, represented by $F_V$. Based on the indicators identified in the prior step, the Global Findex data values (male $V_q^m$ and female $V_q^f$) for each indicator is aggregated:

6 $$\begin{aligned} F_V = \sum_{q=1}^{j}\frac{V_q^f}{V_q^m}-1. \end{aligned}$$

### Network Analysis

This section proposes a network modeling approach to characterize remittance and migration networks [12]. It then introduces tools for analysis of complex networks, such as degree strength and distribution, community formation, and modularity.

#### Global Remittance Network

A flow network is used to represent the flow of remittances between economies. The Global Remittance Network (GRN) is constructed as a directed graph ${\varvec{G}}=(V,E,W)$, where vertices $V$ represent economies, edges $E$ represent remittances flows, and weights $W$ represent remittance values in millions of USD. Remittance flows are constituted as $E=e_{ij}$, where $e_{ij}$ is the directed remittance from sending country $i$ to receiving country $j$. Weights are similarly constituted as $w_{ij}$, representing the total value amount sent from country $i$ to country $j$.

#### Topological Properties

The node degree is the number of edges (remittance flows) of the nodes. For directed networks, degree $k_i$ can be distinguished between in-degree $k_i^{in}$ (number of inward remittances), and out-degree $k_i^{out}$ (number of outward remittances). The node strength is the sum of weights of the node degrees for weighted graphs, calculated from $W$. Node strength can also be distinguished between in-strength $s_i^{in}$ (sum of weights of inward remittances), and out-strength $s_i^{out}$ (sum of weights of outward remittances).

The community structures of the network is detected, wherein nodes in the same community are more strongly connected than nodes in separate communities. The Walktrap algorithm [13] is used, to detect communities in weighted graphs based on short random walks. It assumes that a random walker tends to get trapped in dense parts of a network, i.e., communities. Modularity is commonly used to assess the community structure of a graph. Networks with a high modularity have dense connections between nodes within communities, but sparse connections between nodes in other communities. The form defined by Clauset, Newman, and Moore [14] calculates modularity $Q$ as:

7 $$\begin{aligned} Q=\frac{1}{2E}{\sum }_{ij}\left[ A_{ij}-\frac{k_i k_j}{2E}\right] \delta (C_i,C_j), \end{aligned}$$

where the probability of an edge existing between nodes $i$ and $j$ if connections are made at random but respecting the node degree is $\frac{(k_i k_j)}{2E}$. It calculates the fraction of actual edges found within the same community minus the expected value of a similar network, or $A_{ij}-\frac{(k_i k_j)}{2E}$. $C_i$ is the community to which node i belongs, and $\delta (C_i,C_j) = 1$ if community assignments $C_i$ and $C_j$ are the same. $Q$ is zero if the network’s edges are formed randomly. Nonzero values represent deviations from random formation, with values above 0.3 considered an empirical measure of significant community structure in a network.

#### Global Migration Network

Construction and analysis of the Global Migration Network (GMN) is conducted using the same methodology as the Global Remittance Network. The GMN differs in that the edges represent migration flows between sending and receiving economies, and weights represent the number of migrants.

#### Jaccard Similarity Index

To compare sets of communities across years, and to compare GRN communities with GMN communities, the Jaccard similarity index is used. Community membership for each node $V$ is represented in a binary data matrix, with countries $V$ as rows and communities $N$ as columns. A value of 1 is assigned if a country is a member of community $C_n$, and 0 is assigned if they are not. The steps for calculating the Jaccard index of any two sets (columns n) are:

1. Count the countries which are shared between both communities (where both values are 1)
2. Count the number of countries in both sets, shared and unshared (where values are either 0 or 1)
3. Divide the number of shared members by the total number of members.

The formula is given as:

8 $$\begin{aligned} J(C_n,C_{n'}) = \frac{|C_n \cap C_{n'}|}{|C_n \cup C_{n'}|} = \frac{|C_n \cap C_{n'}|}{|C_n|+|C_{n'}|-|C_n \cap C_{n'}|}, \end{aligned}$$

where J is the Jaccard index, and $C_n$ and $C_{n'}$ are binary data sets 1 and 2, respectively. It measures the similarity of two sets of binary data with a coefficient between 0 and 1. Two sets that share no members would be 0, and sets that share all members would have a coefficient of 1.

#### Bow-tie Structure Analysis

**Fig. 1:**
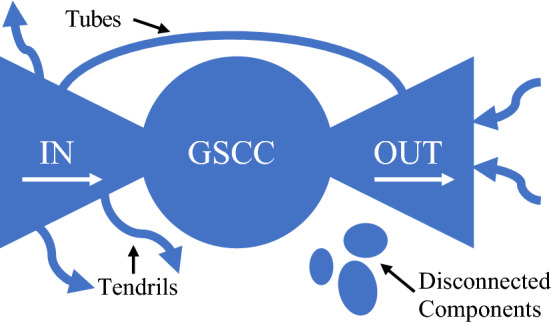
Bow-tie structure. Schematic diagram of the bow-tie structure of a directed network

Bow-tie structures, as illustrated in Fig. 1, have been used to explain the structural behavior of multi-layer networks. A graph G is decomposed into core and peripheral components according to connectivity. All nodes are classified into one of the following components:

- Giant Strongly Connected Component (GSCC): Where any two nodes are reachable through a direct path
- IN Components: Nodes are reachable to the GSCC, but not from the GSCC
- OUT Components: Nodes are reachable from the GSCC, but not to the GSCC
- DISC Components: Nodes are not connected any component

#### Assessing the Gender Gap Between Adjacent Nodes

The correlation of the FIGGS between adjacent nodes is measured. The FIGGSs $F_V$ for each economy $V$ are standardized using the same equation outlined in (3), with $Z_V$ as the new observed value:

9 $$\begin{aligned} Z_V = \frac{F_V-\mu }{\sigma }. \end{aligned}$$

The correlation $H$ is used to examine homophily in the remittance and migration networks. For each remittance- or migration-sending node $i$, we calculate the sum of its standardized FIGGS, $Z_i$, and the standardized FIGGS of all its neighbors, $Z_j$. $Z_j$ is a neighbor of $Z_i$ if its edge, $e_{ij}=1$; lack of an edge is expressed as $e_{ij}=0$. This is sum is then divided by $k_i$, the total number of links $i$ has to other nodes:

10 $$\begin{aligned} H_i = \frac{Z_i + {\sum }_{j=1}^{N}e_{ij}Z_j}{k_i}. \end{aligned}$$

A positive $H$ indicates that similar nodes are more likely to be connected than dissimilar ones in the network. In this case, countries with a higher-than-average FIGGS are likely to be linked to other high FIGGS countries, and nodes with a below-average FIGGS are likely to be linked to other low FIGGS countries. A negative $H$ indicates that linked nodes tend to be dissimilar, with above-average nodes more likely to be linked with below-average ones, and vice versa.

## Results

### Financial Inclusion Gender Gap Score

Table 1 shows the variance explained by each of the first three singular values. After the second, the singular values cease to pick up significant variance. Only first two singular values are considered for this study, as together they capture at least 90% of the variance across both sex-disaggregated datasets.

After measuring the eigenvalues across two dimensions, the results of the “Male” and “Female” sets are compared, and the Z score extracted according to the methodology detailed above. The questions above the threshold $|Z_q^r| = 1$ are identified as the indicators that most contribute to the gender gap. The 10 questions that meet this criterion are presented in Table 2. Thematically, these questions pertain to account ownership, emergency funding, and receiving payments.

With these focus questions identified, the differences in “Male” and “Female” for these questions are extracted. Each economy’s FIGGS is calculated as the sum of their performance across these questions. A lower FIGGS corresponds with less financial inclusion of women. A positive FIGGS indicates that women exceed men in inclusion under these conditions. The ranks and scores for each economy are available in Appendix II.

**Table 1:** Cumulative variance explained by singular values

Dimensions ($r$)	Male (%)	Female (%)
1	87.66	80.47
2	94.47	91.72
3	95.83	94.08

**Table 2:** Extracting the gender gap

	Question	$D_q$	$Z$
Q5	Account	− 0.099	− 6.824
Q230	Borrowed from a financial institution	− 0.360	− 24.632
Q302	Coming up with emergency funds: not possible	− 0.036	− 2.475
Q326	Main source of emergency funds: family or friends	− 0.016	− 1.144
Q338	Main source of emergency funds: money from working	− 0.030	− 2.112
Q350	Main source of emergency funds: loan from a bank, employer, or private lender	− 0.028	− 1.965
Q458	Paid utility bills in the past year	− 0.018	− 1.272
Q478	Received wages in the past year	− 0.256	− 17.520
Q502	Received private sector wages in the past year	− 0.183	− 12.519
Q514	Received public sector wages in the past year	− 0.086	− 5.924

### Remittance and Migration Network Analysis

#### Topological Properties

Key economies are identified based on node degree and strength. Highest ranking economies are made up primarily of large, developed economies (such as US, France, UK, Australia), countries with large populations (India and China), countries with large emigrant populations (such as Mexico and the Philippines), and large migrant populations (such as UAE, Saudi Arabia). A symmetrical relationship is observed between the remittance and migration networks, wherein top remittance receiving economies are also top migrant sending economies and vice versa.

Both remittance and migration networks have asymmetric degree distributions, with many low-degree nodes and fewer high-degree nodes. Excluding the highest-degree countries, countries tend to receive remittances from more countries than they send to, and send migrants to more countries than they receive from. This trend is in line with past findings on degree distributions of remittance and trade networks [7, 15].

**Table 3:** Remittance and migration community sizes, 2010–2017

Remittance				Migration			
	2010	2013	2017		2010	2013	2017
R1	67	5	59	M1	2	2	33
R2	3	29	96	M2	9	69	13
R3	74	59	6	M3	60	13	20
R4	7	16	16	M4	22	27	53
R5	4	13	28	M5	10	11	64
R6	15	82	7	M6	5	2	2
R7	26	7	1	M7	7	53	3
R8	5	2	1	M8	61	3	16
R9	1	1	–	M9	7	9	9
R10	1	–	–	M10	2	2	33
R11	1	–	–	M11	14	15	–
R12	1	–	–	M12	5	3	–
R13	1	–	–	M13	1	1	–
R14	1	–	–	M14	1	1	–
R15	1	–	–	M15	1	–	–
R16	1	–	–	M16	1	–	–
R17	1	–	–	M17	1	–	–
R18	1	–	–	M18	1	–	–
R19	1	–	–	M19	1	–	–
R20	1	–	–	M20	1	–	–
–	–	–	–	M21	1	–	–

**Table 4:** Modularity scores

Year	2010	2013	2017
GRN	0.3578	0.4429	0.4386
GMN	0.5199	0.4887	0.4971

#### Communities

**Fig. 2:**
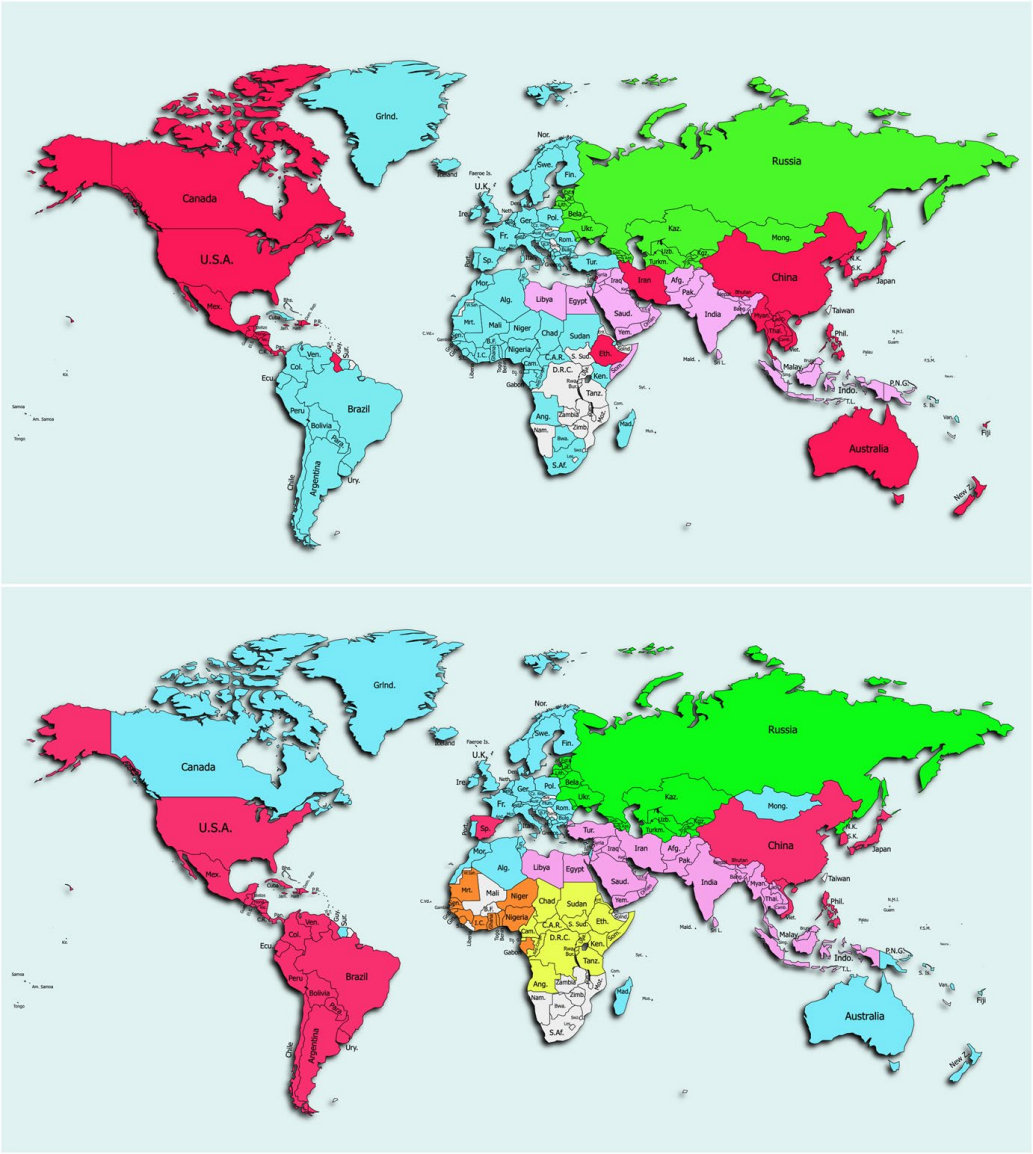
Community Structure of 2017 Global Remittance Network (top) and Global Migration Network (bottom). The colors of the economies correspond with their community membership, and colors are assigned for communities with at least 10 members. In both networks, communities are clearly formed along geographic and economic ties. The migration network is more segmented, as can be observed with the formation of inter-African communities. The migration network also appears to be more driven by cultural and linguistic ties

Community structures represent groups of economies that are more likely to send remittances or migrants between each other than to economies in other groups. Table 3 shows the number of economies making up each community for the years 2010, 2013, and 2017. Communities vary in sizes, but years before 2013 have many single-node communities making up over half of their communities. These single-node communities are primarily small island nations, but also include countries across Africa, Asia, and Europe. From 2013 onward, the gradual integration of communities can be observed, with the communities stabilizing into a smaller number of larger groups.

Figure 2 visualizes the community structures of the remittance network (top) and migration network (bottom) based on 2017 data. The members of each 2017 community are outlined in Appendix III. The GRN is divided into 4 major communities of over 10 economies, while the GMN is divided into 6.

The modularity of each network is measured to assess the community structures, as seen in Table 4. A modularity score of 0 indicates a random formation of communities, and a score of over 0.3 generally indicates a significant community structure. The 2010 remittance has a modularity score of 0.3678, indicating reasonable community structure. This improves to over 0.4 from the year 2013 onwards. The migration network also has a strong community structure, which trends higher than the remittance community scores.

As seen in Fig. 2, these communities are clearly formed along geographic lines. Remittance communities tend to be based on economic and historic (postcolonial) ties. Over 70% of economies are in either the Transatlantic community (R2), comprising most of Europe, Africa, and South America, or the Transpacific community (R1), making up a type of Pacific Rim. The members of major remittance communities are:

- R2 (blue)—96 economies, dominated by South America, Europe, and Africa.
- R1 (red)—59 economies, made up of North America, East Asia, and Oceania.
- R5 (purple)—28 economies, made up of the Middle East, South Asia, and parts of Southeast Asia.
- R4 (green)—16 economies, made up by Russia, Eastern Europe, and Central Asian economies.

The members of major migration communities are:

- M5 (blue)—64 economies, dominated by Europe but including Commonwealth economies, some North African countries, and Mongolia
- M4 (red)—53 economies, made up of the U.S., Central and South America, Spain, and East Asia.
- M1 (purple)—33 economies, consisting of the Middle East, South Asia, and Southeast Asia.
- M3 (yellow)—20 economies, made up of the Central and East Africa.
- M2 (orange)—13 West African economies.
- M8 (green)—16 economies, made up by Russia, Eastern Europe, and Central Asian economies.

The Jaccard Similarity Coefficient is used to compare the makeup of different communities, and the full Jaccard Similarity Indexes are shared in Appendix IV. To determine the stability of the communities over time, the respective GRN and GMN communities are compared on a year-by-year basis for 2010, 2013, and 2017. Between 2010 and 2013, the number of communities in both networks shrink drastically, with all but one of the single-node communities (St. Martin) absorbed into the largest communities. From 2013, the communities stabilize, and a Jaccard Coefficient equaling or nearing 1 becomes the norm. In summary, both remittance and migration networks followed a similar trend of progressive integration and stabilization over the available years.

To determine the relationship between GRN and GMN communities, community membership across networks is compared for the years 2010, 2013, and 2017. Many of the same economies form single-node communities in the 2010 GRN and GMN. As the networks consolidate after 2010, the similarities and differences between the two networks become more apparent. Some communities, such as the community of Middle Eastern and South Asian countries and the community of former Soviet countries, are highly similar with a coefficient of 0.69 and 0.88 respectively. The two largest communities, illustrated in red and blue in Fig. 2, have coefficients of 0.53 and 0.48 respectively, indicating that nearly half the members overlap between the networks.

#### Bow-tie Structure Analysis

A bow-tie structure is not identified with the migration network, as over 99% of GMN nodes are in the GSCC. However the remittance network forms a bow-tie structure. The GSCC, wherein any two nodes are reachable through a direct remittance path, consists of 83.18% of GRN nodes. The IN component, with economies that send but do not receive remittances from the GSCC, makes up 15%. The OUT component, or economies that receive but do not send remittances to the GSCC makes up 1.40%. The distribution of the remittance network shows that a significant majority of economies are actively involved in exchanging remittances. The second largest component is the IN network, indicating the presence of economies that are still excluded from the benefits of migrant remittance inflows.

A similar distribution was observed within major remittance communities. In communities R1 (59 nodes), R2 (96 nodes) and R5 (28 nodes), the GSCC contains roughly 80% of nodes (79.7%, 81.2% and 85.7%, respectively), IN components of roughly 15% (16.9%, 14.6% and 14.3%), and OUT components of 1 or 2 nodes (3.4%, 4.2% and 0%).

#### FIGGS in the Global Remittance and Migration Networks

**Fig. 3:**
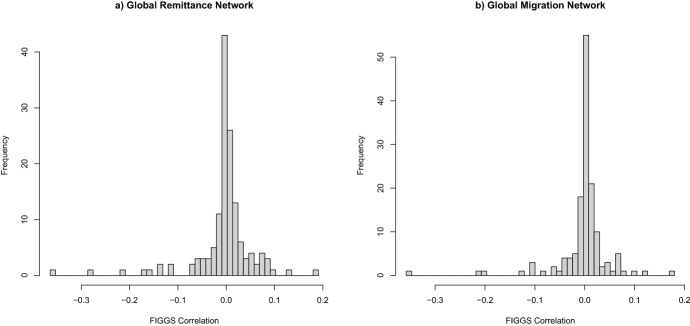
FIGGS Among Adjacent Nodes.The correlation between FIGGS of adjacent nodes are compared for the Global Remittance Network (left) and Global Migration Network (right). There is no observable correlation between the FIGGS of linked nodes

**Table 5:** Average FIGGS by community

Remittance			
Community	Number of nodes	Total FIGGS	Average FIGGS
R1	29	− 10.961	− 0.378
R2	69	− 81.477	− 1.181
R3	4	− 5.808	− 1.452
R4	16	− 19.509	− 1.219
R5	18	− 37.076	− 2.060
R6	6	− 6.473	− 1.079
R7	1	− 2.755	− 2.755

Migration			
Community	Number of nodes	Total FIGGS	Average FIGGS
M1	25	− 42.844	− 1.714
M2	11	− 7.178	− 0.653
M3	11	− 19.785	− 1.799
M4	28	− 15.213	− 0.543
M5	42	− 46.988	− 1.119
M7	3	− 7.065	− 2.355
M8	15	− 17.467	− 1.164
M9	8	− 7.518	− 0.940

**Fig. 4:**
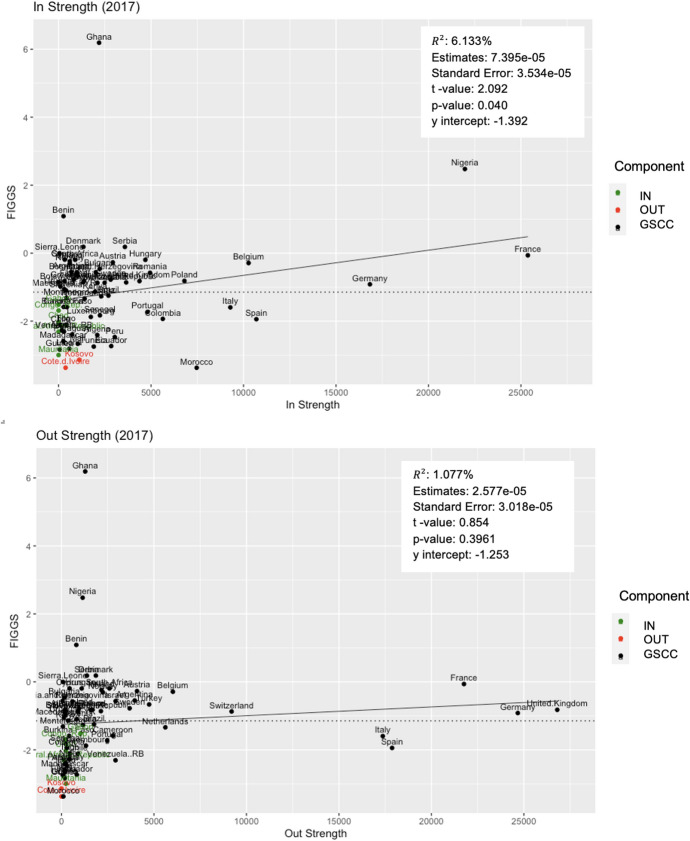
Remittance Community 2 FIGGS and Node Strength. Plots the FIGGS against node strength for Remittance Community 2. Colors show components of the bow-tie structure, with green indicating IN and red OUT components. The solid line is a linear trendline, and the dotted line is the average FIGGS for this community

**Fig. 5:**
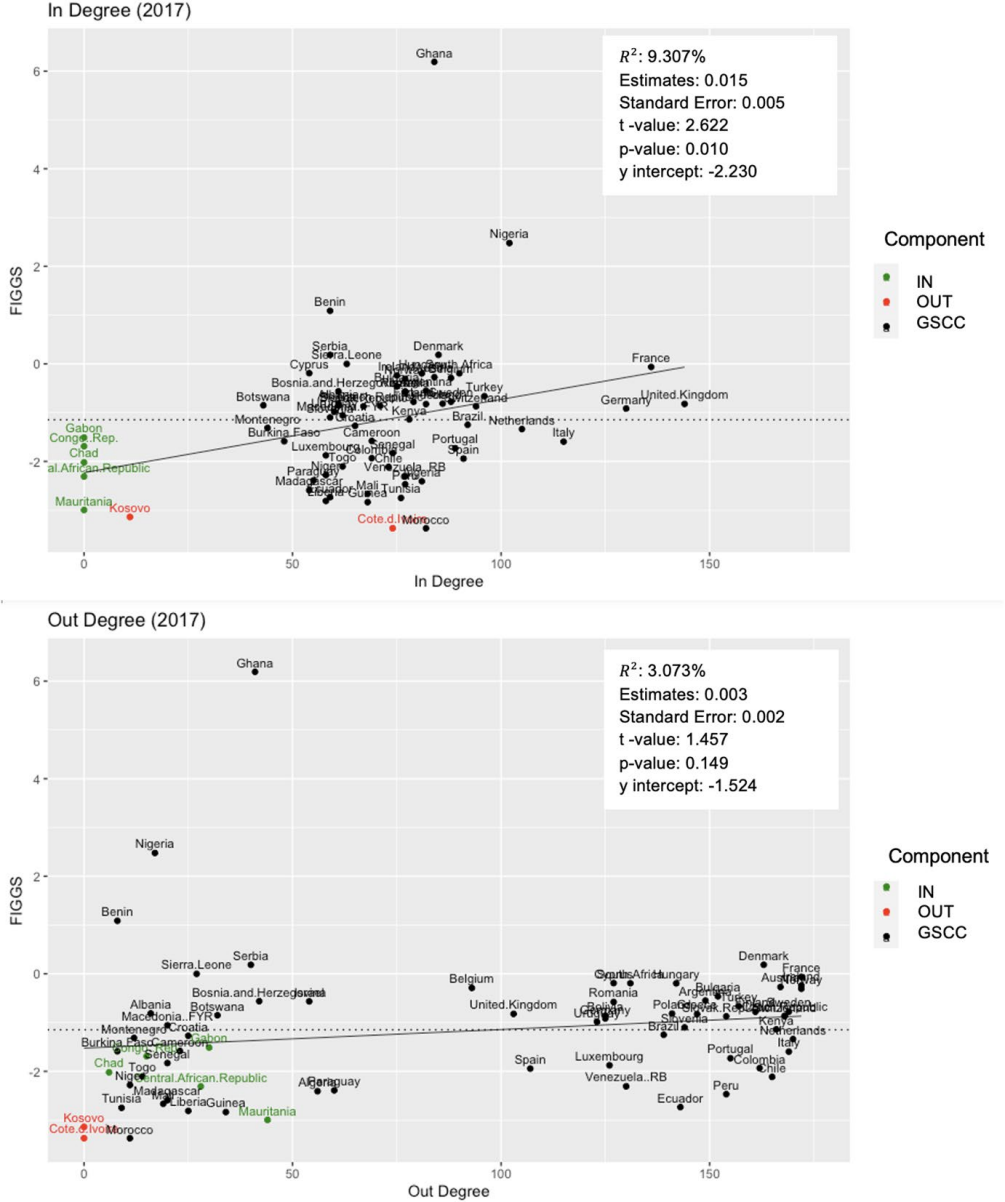
Remittance Community 2 FIGGS and Node Degree. Plots the FIGGS against node degree for Remittance Community 2. Colors show components of the bow-tie structure, with green indicating IN and red OUT components. The solid line is a linear trendline, and the dotted line is the average FIGGS for this community

The correlation of the FIGGS between adjacent nodes is measured. Figure 3 plots the frequency of $H_i$, the sum of the FIGGS of linked nodes divided by the number of links, for the remittance and migration networks. The ratio of positive to negative values is roughly even, with 76:67 in the remittance network and 75:68 in the migration network. Adjacent nodes do not seem to have a correlating gender gap score between them.

FIGGS are calculated for remittance and migration communities. Discrepancies exist in the number of economies included in the Global Findex data (143 economies) and the Bilateral Remittance and Migration Matrices (214 economies). This means that FIGGS are only available for 143 of the 214 economies in the remittance and migration networks.

Table 5 gives the number of nodes with available FIGGS in each community, as well as the aggregated and average scores of each community. Remittance community 8 and migration communities 6 and 10 have no nodes with available FIGGS. Based on available scores, remittance communities 5 and 7 have average scores of less than − 1.5, as do migration communities 1, 3 and 7. These represent small to medium-sized communities made up by a large proportion African, Middle Eastern, and South-Central Asian countries.

The FIGG score is also applied to the remittance network bow-tie structure analysis. The peripheral IN, OUT, and DISC components are made up of many smaller island nations which are not included in the Global Findex dataset. Based on the available scores, the component with the highest average FIGGS is the GSCC (− 1.045), followed by the IN component (– 1.976) and OUT component (− 3.255). The DISC component does not have a score available. Of the remittance communities, the largest of R2 has FIGGS available for the GSCC, IN, and OUT components. The average score for each component is calculated as GSCC (− 1.039), IN (− 2.106), and OUT (− 3.255).

To study an economy’s relative gender gap within its community, the relationship between an economy’s FIGGS and node attributes is further explored for the largest community, Remittance Community 2 (R2). The FIGGS is plotted against node strength in Fig. 4 and node degree in Fig. 5. Intuitively, peripheral nodes have a comparatively smaller role in the network, both in terms of remittance partners (in- and out-degree) and remittance value (in- and out-strength). Economies in the IN and OUT components fall below the mean FIGGS (dotted line). A slight positive relationship between node degree/strength and FIGGS is also observed, with in-strength and in-degree as especially statistically significant variables.

## Discussion

### Financial Inclusion Gender Gap Score

The 2017 Global Findex dataset includes 45 questions that can be disaggregated by gender. The methodology presented in this study highlight the 10 questions that are most statistically significant in explaining the gender gap. Thematically, these questions pertain to account ownership, emergency funding, and receiving payments.

Several questions relating to receiving payments are included (Q478 Received wages in the past year; Q502 Received private sector wages in the past year; Q514 Received public sector wages in the past year). Some national and regional studies have found women are also more likely to receive informal wages or use informal intermediary services [16, 17]. The gap in these variables may also be determined by factors outside the financial sector, such as the gender gaps in labor force participation [10] or legal discrimination [4]. The role of government payments as a catalyst for financial inclusion - including for women specifically—has also been lauded. Social assistance and government payment programs have been a cornerstone of national financial inclusion policies in countries that have made tremendous progress in reducing the gender gap, such as Mexico and India. The lack of payments through formal channels has further consequences in banking penetration, as it prevents access to extended services such as to credit and borrowing.

The inclusion of emergency funding variables (Q302 Coming up with emergency funds: not possible; Q326 Main source of emergency funds: family and friends; Q326 Main source of emergency funds: loan from a bank, employer, or private lender) is significant considering the abundance of reports and studies on the role of migrant remittances in mitigating shocks. Migrant remittances sent back to home countries are know to be a lifeline when emergencies such as natural disasters [18] or health crises [19] occur, and act as a source of insurance outside the household [20]. Migrant remittances are also understood as a tool for asset-building and risk management [21], contributing to long-term disaster resilience. The inclusion of these questions shows that women have considerably less access to formal credit, and are more reliant on familial and communal relationships to see themselves through in the case of an emergency. They therefore stand to benefit substantially from being better able to receive migrant remittances.

### Financial Inclusion Gender Gap: Community Level

Community formation is closely related between remittance and migration networks, with structures reflecting economic, political, and social conditions. The topology and structures of the networks change across the years studied, with gradual consolidation into major distinct communities by 2017. The migration network structure remains slightly less integrated than the remittance network, with a total of six community of over ten economies where the remittance network has four. While geographic proximity is a clear explanatory factor in community formation across both networks, most communities are supra- or sub-regional. The networks’ community structure, therefore, provides a more nuanced understanding of remittance and migration linkages than general regional classification might.

The remittance network holds larger and more integrated communities, whose community structure can be seen as more economically driven. The largest communities are those making up the Pacific (59 economies) and Atlantic (96 economies) rim. The migration network has a higher modularity but is more segmented due to the presence of significant regional African communities—a West African and a Central/East African community. The basis for the migration network also appears to shift based on linguistic and cultural ties. In 2017, a new major community emerges as an independent Western African community. The U.K. shifts from the transpacific community to the European community in 2013, and Canada, Australia, and New Zealand follow together by 2017. It is possible that as English-speaking members of the Commonwealth, these four countries share close migration relationships. Spain moves from the European community to the Pacific block in 2017, perhaps explained by its shared language and history with South America. These findings are in line with existing literature on the positive impact of postcolonial ties and common language on migration flows [22]. Southeast Asia also shifts to the Middle East and South Asia community, with the addition of countries like Thailand, Myanmar, Cambodia, and Laos. Interestingly, Mongolia is also part of the European community across 3 years and does not join the block of former Soviet countries.

In calculating the average FIGGS of remittance and migration communities, the relative significance of a gender gap is revealed. Across both networks, communities with a lower average FIGGS were focused in certain regions —  namely the Middle Eastern/South Asian communities of R5 (− 2.060) and M1 (− 1.714). R5 and M1 share a Jaccard Similarity Coefficient of 0.69, having many lower scoring economies in common. The independent African migration communities—such as M3 (average FIGGS of − 1.799), and M7 (− 2.355)—have lower average FIGGS, however member economies are integrated into the large Transatlantic community (R2) in the remittance network. Existing literature focuses heavily on Sub-Sharan Africa, the Middle East, and South Asia [10, 16, 23], and the results of this study affirms the importance of these regions as areas of focus.

The lower-scoring communities are also small- to mid-sized, and the largest communities trend toward higher average FIGGS. Compared to remittance communities, migration communities are more clearly divided into higher average and lower average FIGGS communities. The remittance network has two communities with an average FIGGS of less than − 2.0, where the migration network has three. The remittance network has one community with an average FIGGS above − 0.5, where the migration network has three. Most remittance communities (four out of seven) have an average FIGGS in the range between − 1.0 and − 2.0, while only two of nine migration communities are in the same range. This indicates that the determinants of migration community formation—seemingly driven by social and cultural factors—have more in common with the determinants of a financial inclusion gender gap.

### Financial Inclusion Gender Gap: Country Level

Over 90% of economies have a negative FIGGS, confirming an extensive financial inclusion gender gap in favor of men. However, fourteen countries are found to have a positive FIGGS, meaning women make up a higher percentage of benefactors of the services discussed above. Of these, the top five highest scoring countries were Vietnam (25.537), Ghana (6.191), Myanmar (3.718), Haiti (3.173), and Nigeria (2.477). In all five cases, their high FIGGS are driven solely by women outperforming men in Q350 (“Main source of emergency funds: loan from a bank, employer, or private lender”), and their scores in other areas are average. This indicates segmentation within these societies, where although financial access remains limited for women overall, women who are more financial included are able to proactively draw from highly sophisticated financial services.

An economy’s relative FIGGS within its community is investigated using bow-tie structure analysis. In calculating the average FIGGS of each component, it is revealed that an economy’s position within the bow-tie predicts its relative gender gap performance within the network. Economies that are strongly connected have a higher average rating (GSCC average of − 1.034) than IN component economies (− 1.976), and OUT component economies scored worst of all (− 3.255).

Another notable finding is the divergence of remittance and migration network structures in their bow-tie structures. Intuitively, the IN-component economies of the migration network should correspond with the OUT component economies of the remittance network and vice versa. This would confirm the symmetrical relationship between migration and remittances, where a migrant going from country $i$ to country $j$ would send remittances back from country $j$ to country $i$ [5]. However, a bow-tie structure was not observed in the migration network, as 99% of nodes formed the GSCC, leaving 1% for the IN component.

While the IN component economies of the migration network match the OUT component economies of the remittance network (Kosovo and St. Martin), the lack of OUT component in the migrant network (economies that do have emigrants to the GSCC) is somewhat unexpected. It implies that economies in the remittance IN component send migrants to the GSCC but are not receiving significant remittance from them. These economies include a large share of the economies and territories that are not captured in the Findex database. In terms of community membership, these smaller IN component economies are found primarily in the major communities 1 and 2. Their membership appears based largely on historic relationships, such as the inclusion of American Samoa, Guam, and Puerto Rico in the same community as the U.S. (R1), or Andorra and Monaco with the European community (R2). The remaining economies are focused in R5 and include many African countries with a lower average FIGGS performance, such as Libya (− 4.121) and Mauritania (− 2.996). Their presence in the remittance IN component therefore corroborates their low financial inclusion performance as identified in the FIGGS analysis. It may indicate a lack of remittance-receiving infrastructure in these economies, or the dependence of emigrants on informal remittance channels to send money home.

This study also found a positive relationship between relative gender gap performance within its community and node attributes such as degree and weighted degree strength. Economies with a smaller number of partner economies (in- and out-degrees) and remittance and migration output (in- and out-strength) trend toward a lower FIGGS, as most clearly observed in the dominant remittance community 2 (see Fig. 2). However, these results were skewed in the case of the top two in-strength countries of India (USD 69 bio) and China (USD 64 bio), who receive remittances valuing nearly twice the next highest in-strength country (Philippines, USD 33 bio) and far surpass other economies in in-strength due to their enormous populations of outward migrants who remit home [24]. These countries have relatively low FIGGS of − 2.077 and − 1.920, respectively. Although limitations in the FIGGS data prevented corroboration across the full network, we can infer that economies with a smaller role the network exhibit a larger gender gap. This finding further supports the application of network analysis in understanding the influence of globalization on development indicators.

## Conclusion

Ensuring equitable access to financial services is an important realization of international development goals. In recent years, the rise in global migration and subsequent rise in remittances by migrants has opened up the potential of migrant remittances as a catalyst for greater financial inclusion. Research on this topic has been dominated by local-level and country-level studies, which are not reflective of the global linkages that exist in human-capital flows. The novelty of our approach is therefore in the integration of a network perspective to the study of financial inclusion. We propose an original multimodal analysis on multiple global datasets to determine the main contributors of the gender gap in financial inclusion, and investigate the predictive powers of remittance and migration network features on regional and national gender gaps.

This study builds on literature on the use of statistical techniques to measure financial inclusion. It applied SVD to the gender-disaggregated 2017 Global Findex dataset to analyze the global gender gap and found that lack of access to emergency funding and receiving payments were especially significant contributors. These results corroborate past findings that women are less likely to use formal financial products and services and are more reliant on informal intermediaries for loans and payments. Over 90% of nodes were found to have a negative FIGGS, reaffirming that the gender imbalance remains a pervasive global issue.

This study compared community structures of global remittance and migration networks for years between 2010 and 2017. A gradual integration of both networks was observed across the available years, and the emergence of several major geographically linked communities was observed. The remittance community formation was found to be more economically driven, while the migration community reflected more political and sociocultural influences. In assessing the average FIGGS for remittance and migration networks, it was revealed that women’s financial inclusion is poorest among African, Middle Eastern, and South Asian communities. The migration communities were found to have higher and lower average scores, meaning the community membership included more similarly scoring economies. It can be inferred that the determinants of migration community formation may have more in common with the determinants of a financial inclusion gender gap.

At the country level, economies with smaller roles in the network were found to have a more sizeable gender gap. This finding was consistent across several indicators, including node degree and strength, and bow-tie component membership. That economies that are less closely linked by remittance and migration relationships were more likely to have a gender gap issue speaks to the potential of migrant remittances in fostering women’s financial inclusion.

In conclusion, the results of this study support the understanding of migrant remittances as a key to addressing the gender gap in financial inclusion. The country-level network analysis confirmed that economies that are less integrated in migration and remittance linkages have a greater gender gap. Community-level analysis indicated the regions that have a more substantial gender gap issue, with the gender gap more predicated on the social and cultural leaning determinants of migration communities. Although this research focuses on migration and remittance data, an area for continued research is how other sociocultural and economic factors may enhance our understanding of national and regional FIGGS. Future study will, therefore, incorporate other global economic data to expand the predictive capability of remittance and migration networks on the state of financial inclusion.

## Appendix I: 2017 Global Findex Gender-Disaggregated Questions

**Table:**

Question number	Question
1	Account
13	Financial institution account
44	Used the internet to pay bills in the past year
56	Used the internet to pay bills or to buy something online in the past year
68	Used the internet to buy something online in the past year
82	Saved to start, operate, or expand a farm or business
94	Saved for old age
106	Saved at a financial institution
118	Saved using a savings club or a person outside the family
142	Saved any money in the past year
154	Outstanding housing loan
166	Debit card ownership
178	Borrowed for health or medical purposes
190	Borrowed to start, operate, or expand a farm or business, female
226	Borrowed from a financial institution
238	Borrowed from a financial institution or used a credit card
250	Borrowed from family or friends
262	Borrowed from a savings club
274	Borrowed any money in the past year,
286	Coming up with emergency funds: possible
298	Coming up with emergency funds: not possible
310	Main source of emergency funds: savings
322	Main source of emergency funds: family or friends
334	Main source of emergency funds: money from working
346	Main source of emergency funds: loan from a bank, employer, or private lender
358	Main source of emergency funds: sale of assets
370	Main source of emergency funds: other
382	Sent or received domestic remittances in the past year, female
394	Received domestic remittances in the past year
430	Sent domestic remittances in the past year
454	Paid utility bills in the past year
474	Received wages in the past year
498	Received private sector wages in the past year
510	Received public sector wages in the past year
560	Received government transfers in the past year, female
578	Received a public sector pension in the past year
606	Received payments for agricultural products in the past year
628	Received payments from self-employment in the past year
651	Used a mobile phone or the internet to access an account
694	No deposit and no withdrawal from an account in the past year
717	Received government payments in the past year
729	Made or received digital payments in the past year
741	Made digital payments in the past year
753	Received digital payments in the past year
765	Mobile money account

Question numbers correspond with their numbers in the original index

## Appendix II: 2017 FIGGS Rankings

**Table:**

Rank	Country	FIGGS	Rank	Country	FIGGS
1	Vietnam	25.537	72	Zambia	− 1.455
2	Ghana	6.191	73	Gabon	− 1.512
3	Myanmar	3.718	74	Panama	− 1.547
4	Haiti	3.173	75	Tanzania	− 1.558
5	Nigeria	2.477	76	Cameroon	− 1.582
6	Singapore	1.632	77	Armenia	− 1.586
7	Benin	1.087	78	Burkina Faso	− 1.588
8	Estonia	0.348	79	Italy	− 1.596
9	Lithuania	0.279	80	Guatemala	− 1.632
10	Denmark	0.185	81	Iran	− 1.636
11	Serbia	0.184	82	Congo, Rep.	− 1.690
12	Moldova	0.140	83	Ukraine	− 1.706
13	Korea, Rep.	0.125	84	Kazakhstan	− 1.726
14	Sierra Leone	− 0.001	85	Cambodia	− 1.729
15	Lesotho	− 0.005	86	Portugal	− 1.731
16	France	− 0.063	87	Honduras	− 1.756
17	Sri Lanka	− 0.096	88	Kyrgyz Rep.	− 1.803
18	Rwanda	− 0.103	89	Senegal	− 1.830
19	New Zealand	− 0.158	90	Uganda	− 1.842
20	Philippines	− 0.189	91	Dominican Rep.	− 1.859
21	Cyprus	− 0.191	92	Trinidad &Tobago	− 1.869
22	South Africa	− 0.194	93	Luxembourg	− 1.876
23	Hungary	− 0.196	94	Turkmenistan	− 1.884
24	Ireland	− 0.238	95	Tajikistan	− 1.898
25	Austria	− 0.271	96	China	− 1.920
26	Belgium	− 0.290	97	Colombia	− 1.934
27	Norway	− 0.308	98	Spain	− 1.944
28	Russia	− 0.324	99	Malaysia	− 1.958
29	Belarus	− 0.326	100	Zimbabwe	− 1.982
30	Bulgaria	− 0.464	101	Mexico	− 1.999
31	Argentina	− 0.549	102	Chad	− 2.020
32	Bosnia-Herzegovina	− 0.562	103	Mongolia	− 2.042
33	Israel	− 0.566	104	India	− 2.077
34	Romania	− 0.577	105	Togo	− 2.104
35	Turkey	− 0.664	106	Chile	− 2.114
36	Sweden	− 0.778	107	Bahrain	− 2.131
37	Finland	− 0.778	108	Nicaragua	− 2.184
38	Albania	− 0.812	109	Iraq	− 2.262
39	Poland	− 0.816	110	Niger	− 2.281
40	United Kingdom (UK)	− 0.822	111	Jordan	− 2.297
41	Greece	− 0.826	112	Utd.Arab Emirates (UAE)	− 2.303
42	Australia	− 0.838	113	South Sudan	− 2.305
43	Botswana	− 0.850	114	Venezuela	− 2.308
44	Bolivia	− 0.856	115	Central African Rep.	− 2.310
45	Czech Rep.	− 0.861	116	Paraguay	− 2.391
46	Namibia	− 0.865	117	Algeria	− 2.408
47	Canada	− 0.870	117	Peru	− 2.468
48	Switzerland	− 0.871	118	Ethiopia	− 2.481
49	Slovak Rep.	− 0.872	119	Madagascar	− 2.592
50	Azerbaijan	− 0.902	120	Costa Rica	− 2.648
51	Germany	− 0.916	121	Mali	− 2.667
52	Mozambique	− 0.919	122	Egypt	− 2.706
53	Uruguay	− 0.984	123	Bangladesh	− 2.716
54	Macedonia	− 1.057	124	Ecuador	− 2.734
55	Nepal	− 1.083	125	Tunisia	− 2.749
56	Slovenia	− 1.099	126	Congo, Dem. Rep.	− 2.755
57	Thailand	− 1.125	127	Saudi Arabia	− 2.774
58	Kuwait	− 1.131	128	Afghanistan	− 2.786
59	Kenya	− 1.140	129	Liberia	− 2.811
60	Georgia	− 1.168	130	Guinea	− 2.835
61	Malawi	− 1.248	131	Mauritius	− 2.858
62	Latvia	− 1.249	132	Lebanon	− 2.895
63	Brazil	− 1.251	133	Hong Kong	− 2.948
64	Croatia	− 1.271	134	Malta	− 2.970
65	El Salvador	− 1.284	135	Mauritania	− 2.996
66	United States (US)	− 1.312	136	Kosovo	− 3.139
67	Montenegro	− 1.314	137	Pakistan	− 3.365
68	Netherlands	− 1.338	138	Cote d’Ivoire	− 3.371
69	Japan	− 1.371	139	Morocco	− 3.372
70	Lao PDR	− 1.437	140	West Bank & Gaza	− 3.456
71	Indonesia	− 1.445	141	Uzbekistan	− 3.665
			143	Libya	− 4.121

FIGGS for all economies are ranked from highest to lowest. Positive values imply women are more financially included, and lower scores indicate a higher rate of exclusion

## Appendix III: Remittance and Migration Community Membership

**Remittance Community 1**

**Table:**

Rank	Country	FIGGS
1	Vietnam	25.537
2	Myanmar	3.718
3	Haiti	3.173
4	Korea, Rep.	0.125
5	New Zealand	− 0.158
6	Philippines	− 0.189
7	Australia	− 0.838
8	Canada	− 0.870
9	Thailand	− 1.125
10	El Salvador	− 1.284
11	US	− 1.312
12	Japan	− 1.371
13	Lao PDR	− 1.437
14	Panama	− 1.547
15	Guatemala	− 1.632
16	Iran	− 1.636
17	Cambodia	− 1.729
18	Honduras	− 1.756
19	Dominican Rep.	− 1.859
20	Trinidad &Tobago	− 1.869
21	China	− 1.920
22	Mexico	− 1.999
23	Nicaragua	− 2.184
24	Ethiopia	− 2.481
25	Costa Rica	− 2.648
26	Mauritius	− 2.858
27	Lebanon	− 2.895
28	Hong Kong	− 2.948
29	Malta	− 2.970

**Remittance Community 2 **

**Table:**

Rank	Country	FIGGS
1	Ghana	6.191
2	Nigeria	2.477
3	Benin	1.087
4	Denmark	0.185
5	Serbia	0.184
6	Sierra Leone	− 0.001
7	France	− 0.063
8	Cyprus	− 0.191
9	South Africa	− 0.194
10	Hungary	− 0.196
11	Ireland	− 0.238
12	Austria	− 0.271
13	Belgium	− 0.290
14	Norway	− 0.308
15	Bulgaria	− 0.464
16	Argentina	− 0.549
17	Bosnia-Herzegovina	− 0.562
18	Israel	− 0.566
19	Romania	− 0.577
20	Turkey	− 0.664
21	Sweden	− 0.778

**Remittance Community 2**

**Table:**

Rank	Country	FIGGS
22	Finland	− 0.778
23	Albania	− 0.812
24	Poland	− 0.816
25	UK	− 0.822
26	Greece	− 0.826
27	Botswana	− 0.850
28	Bolivia	− 0.856
29	Czech Rep.	− 0.861
30	Switzerland	− 0.871
31	Slovak Rep.	− 0.872
32	Germany	− 0.916
33	Uruguay	− 0.984
34	Macedonia	− 1.057
35	Slovenia	− 1.099
36	Kenya	− 1.140
37	Brazil	− 1.251
38	Croatia	− 1.271
39	Montenegro	− 1.314
40	Netherlands	− 1.338
41	Gabon	− 1.512
42	Cameroon	− 1.582
43	Burkina Faso	− 1.588
44	Italy	− 1.596
45	Congo Rep.	− 1.690
46	Portugal	− 1.731
47	Senegal	− 1.830
48	Luxembourg	− 1.876
49	Colombia	− 1.934
50	Spain	− 1.944
51	Chad	− 2.020
52	Togo	− 2.104
53	Chile	− 2.114
54	Niger	− 2.281
55	Venezuela	− 2.308
56	Central African Rep.	− 2.310
57	Paraguay	− 2.391
58	Algeria	− 2.408
59	Peru	− 2.468
60	Madagascar	− 2.592
61	Mali	− 2.667
62	Ecuador	− 2.734
63	Tunisia	− 2.749
64	Liberia	− 2.811
65	Guinea	− 2.835
66	Mauritania	− 2.996
67	Kosovo	− 3.139
68	Cote d’Ivoire	− 3.371
69	Morocco	− 3.372

**Remittance Community 3**

**Table:**

Rank	Country	FIGGS
1	Rwanda	− 0.103
2	Tanzania	− 1.558
3	Uganda	− 1.842
4	South Sudan	− 2.305

**Remittance Community 4 **

**Table:**

Rank	Country	FIGGS
1	Estonia	0.348
2	Lithuania	0.279
3	Moldova	0.140
4	Russia	− 0.324
5	Belarus	− 0.326
6	Azerbaijan	− 0.902
7	Georgia	− 1.168
8	Latvia	− 1.249
9	Armenia	− 1.586
10	Ukraine	− 1.706
11	Kazakhstan	− 1.726
12	Kyrgyz Rep.	− 1.803
13	Turkmenistan	− 1.884
14	Tajikistan	− 1.898
15	Mongolia	− 2.042
16	Uzbekistan	− 3.665

**Remittance Community 5 **

**Table:**

Rank	Country	FIGGS
1	Singapore	1.632
2	Sri Lanka	− 0.096
3	Nepal	− 1.083
4	Kuwait	− 1.131
5	Indonesia	− 1.445
6	Malaysia	− 1.958
7	India	− 2.077
8	Bahrain	− 2.131
9	Iraq	− 2.262
10	Jordan	− 2.297
11	UAE	− 2.303
12	Egypt	− 2.706
13	Bangladesh	− 2.716
14	Saudi Arabia	− 2.774
15	Afghanistan	− 2.786
16	Pakistan	− 3.365
17	West Bank &Gaza	− 3.456
18	Libya	− 4.121

**Remittance Community 6**

**Table:**

Rank	Country	FIGGS
1	Lesotho	− 0.005
2	Namibia	− 0.865
3	Mozambique	− 0.919
4	Malawi	− 1.248
5	Zambia	− 1.455
6	Zimbabwe	− 1.982

**Remittance Community 7**

**Table:**

Rank	Country	FIGGS
1	Congo, Dem. Rep.	− 2.755

**Migration Community 1 **

**Table:**

Rank	Country	FIGGS
1	Myanmar	3.718
2	Singapore	1.632
3	Sri Lanka	− 0.096
4	Turkey	− 0.664
5	Nepal	− 1.083
6	Thailand	− 1.125
7	Kuwait	− 1.131
8	Lao PDR	− 1.437
9	Indonesia	− 1.445
10	Iran	− 1.636
11	Cambodia	− 1.729
12	Malaysia	− 1.958
13	India	− 2.077
14	Bahrain	− 2.131
15	Iraq	− 2.262
16	Jordan	− 2.297
17	UAE	− 2.303
18	Egypt	− 2.706
19	Bangladesh	− 2.716
20	Saudi Arabia	− 2.774
21	Afghanistan	− 2.786
22	Lebanon	− 2.895
23	Pakistan	− 3.365
24	West Bank & Gaza	− 3.456
25	Libya	− 4.121

**Migration Community 2 **

**Table:**

Rank	Country	FIGGS
1	Ghana	6.191
2	Nigeria	2.477
3	Benin	1.087
4	Sierra.Leone	− 0.001
5	Gabon	− 1.512
6	Senegal	− 1.830
7	Togo	− 2.104
8	Niger	− 2.281
9	Guinea	− 2.835
10	Mauritania	− 2.996
11	Cote.d.Ivoire	− 3.371

**Migration Community 3**

**Table:**

Rank	Country	FIGGS
1	Rwanda	− 0.103
2	Kenya	− 1.140
3	Tanzania	− 1.558
4	Cameroon	− 1.582
5	Congo Rep.	− 1.690
6	Uganda	− 1.842
7	Chad	− 2.020
8	South Sudan	− 2.305
9	Central African Rep.	− 2.310
10	Ethiopia	− 2.481
11	Congo Dem. Rep.	− 2.755

**Migration Community 4**

**Table:**

Rank	Country	FIGGS
1	Vietnam	25.537
2	Haiti	3.173
3	Korea Rep.	0.125
4	Philippines	− 0.189
5	Argentina	− 0.549
6	Bolivia	− 0.856
7	Uruguay	− 0.984
8	Brazil	− 1.251
9	El Salvador	− 1.284
10	US	− 1.312
11	Japan	− 1.371
12	Panama	− 1.547
13	Guatemala	− 1.632
14	Honduras	− 1.756
15	Dominican Rep.	− 1.859
16	Trinidad & Tobago	− 1.869
17	China	− 1.920
18	Colombia	− 1.934
19	Spain	− 1.944
20	Mexico	− 1.999
21	Chile	− 2.114
22	Nicaragua	− 2.184
23	Venezuela	− 2.308
24	Paraguay	− 2.391
25	Peru	− 2.468
26	Costa Rica	− 2.648
27	Ecuador	− 2.734
28	Hong Kong	− 2.948

**Migration Community 5**

**Table:**

Rank	Country	FIGGS
1	Denmark	0.185
2	Serbia	0.184
3	France	− 0.063
4	Cyprus	− 0.191
5	Hungary	− 0.196
6	Ireland	− 0.238
7	Austria	− 0.271
8	Belgium	− 0.290
9	Norway	− 0.308
10	Bulgaria	− 0.464
11	Bosnia-Herzegovina	− 0.562
12	New Zealand	− 0.158
13	Israel	− 0.566
14	Romania	− 0.577
15	Sweden	− 0.778
16	Finland	− 0.778
17	Albania	− 0.812
18	Australia	− 0.838
19	Canada	− 0.870
20	Poland	− 0.816
21	UK	− 0.822
22	Greece	− 0.826
23	Czech Rep.	− 0.861
24	Switzerland	− 0.871
25	Slovak Rep.	− 0.872
26	Germany	− 0.916
27	Macedonia	− 1.057
28	Slovenia	− 1.099
29	Croatia	− 1.271

**Migration Community 5**

**Table:**

Rank	Country	FIGGS
30	Montenegro	− 1.314
31	Netherlands	− 1.338
32	Italy	− 1.596
33	Portugal	− 1.731
34	Luxembourg	− 1.876
35	Algeria	− 2.408
36	Madagascar	− 2.592
37	Tunisia	− 2.749
38	Mauritius	− 2.858
39	Mongolia	− 2.042
40	Kosovo	− 3.139
41	Morocco	− 3.372
42	Malta	− 2.970

**Migration Community 7**

**Table:**

Rank	Country	FIGGS
1	Burkina Faso	− 1.588
2	Mali	− 2.667
3	Liberia	− 2.811

**Migration Community 8 **

**Table:**

Rank	Country	FIGGS
1	Estonia	0.348
2	Lithuania	0.279
3	Moldova	0.140
4	Russian	− 0.324
5	Belarus	− 0.326
6	Azerbaijan	− 0.902
7	Georgia	− 1.168
8	Latvia	− 1.249
9	Armenia	− 1.586
10	Ukraine	− 1.706
11	Kazakhstan	− 1.726
12	Kyrgyz Republic	− 1.803
13	Turkmenistan	− 1.884
14	Tajikistan	− 1.898
15	Uzbekistan	− 3.665

**Migration Community 9 **

**Table:**

Rank	Country	FIGGS
1	Lesotho	− 0.005
2	South Africa	− 0.194
3	Botswana	− 0.850
4	Namibia	− 0.865
5	Mozambique	− 0.919
6	Malawi	− 1.248
7	Zambia	− 1.455
8	Zimbabwe	− 1.982

Countries in each remittance and migration community are listed in order of highest to lowest FIGGS

## Appendix IV: Jaccard Similarity Coefficients

**Remittance Communities**

**a) 2010 vs 2013 **

**Table:**

		**2010**									
		R1	R2	R3	R4	R5	R6	R7	R8	R9	R10
		(67)	(3)	(74)	(7)	(4)	(15)	(26)	(5)	(1)	(1)
	R1 (5)	0.01	0	0	0	0	0.05	0	0.25	0	0
	R2 (29)	0.07	0	0.08	0	0	0.02	0.27	0	0	0
	R3 (59)	0.34	0.01	0.07	0.01	0.03	0.04	0.06	0.01	0	0
**2013**	R4 (16)	0.02	0	0.02	0.27	0	0.19	0.05	0	0	0
	R5 (13)	0.03	0.14	0.08	0	0	0	0.02	0	0	0
	R6 (82)	0.15	0	0.37	0.01	0.02	0.03	0.05	0.02	0.01	0.01
	R7 (7)	0.01	0	0.08	0	0	0	0	0	0	0
	R8 (2)	0	0	0	0	0	0.13	0	0	0	0
	R9 (1)	0.01	0	0	0	0	0	0	0	0	0

**2010 vs 2013**

**Table:**

		**2010**									
		R11	R12	R13	R14	R15	R16	R17	R18	R19	R20
		(1)	(1)	(1)	(1)	(1)	(1)	(1)	(1)	(1)	(1)
	R1 (5)	0.2	0	0	0	0	0	0	0	0	0
	R2 (29)	0	0	0	0	0	0	0.03	0	0	0
	R3 (59)	0	0	0.01	0	0.01	0.01	0	0	0.01	0.01
**2013**	R4 (16)	0	0	0	0	0	0	0	0	0	0
	R5 (13)	0	0	0	0	0	0	0	0	0	0
	R6 (82)	0	0.01	0	0.01	0	0	0	0.01	0	0
	R7 (7)	0	0	0	0	0	0	0	0	0	0
	R8 (2)	0	0	0	0	0	0	0	0	0	0
	R9 (1)	0	0	0	0	0	0	0	0	0	0

**b) 2013 vs 2017 **

**Table:**

		**2013**								
		R1	R2	R3	R4	R5	R6	R7	R8	R9
		(5)	(29)	(59)	(16)	(13)	(82)	(7)	(2)	(1)
	R1 (59)	0	0	0.93	0	0	0	0	0.03	0
	R2 (96)	0	0	0.01	0	0.13	0.81	0	0	0
	R3 (6)	0.57	0	0	0	0	0.02	0	0	0
**2017**	R4 (16)	0	0	0	1	0	0	0	0	0
	R5 (28)	0	0.96	0	0	0	0	0	0	0
	R6 (7)	0	0	0	0	0	0	1	0	0
	R7 (1)	0.2	0	0	0	0	0	0	0	0
	R8 (1)	0	0	0	0	0	0	0	0	1

**Migration Communities**

**a) 2010 vs 2013 **

**Table:**

		**2010**									
		R1	R2	R3	R4	R5	R6	R7	R8	R9	R10
		(2)	(9)	(60)	(22)	(10)	(5)	(7)	(61)	(7)	(2)
	R1 (2)	0	0	0.01	0	0	0	0	0.01	0	0
	R2 (69)	0.02	0	0.38	0.05	0	0.01	0.02	0.15	0.01	0.01
	R3 (13)	0	0	0.01	0	0	0	0	0.02	0.11	0
	R4 (27)	0	0.02	0.08	0.28	0.02	0	0.03	0.04	0	0
	R5 (11)	0	0	0.02	0	0.31	0.06	0.12	0.01	0	0
	R6 (2)	0	0	0.01	0	0	0	0.12	0	0	0
**2013**	R7 (53)	0	0.03	0.06	0.02	0.01	0.01	0.01	0.43	0	0
	R8 (3)	0	0.09	0	0.04	0	0	0	0	0	0.25
	R9 (9)	0	0.38	0.01	0	0	0.07	0	0.01	0	0
	R10 (5)	0	0	0	0.03	0	0	0	0	0.5	0
	R11 (15)	0	0	0.04	0.05	0	0.05	0	0	0	0
	R12 (3)	0	0	0	0	0.08	0	0	0.01	0	0
	R13 (1)	0	0	0	0	0	0	0	0	0	0
	R14 (1)	0	0	0.01	0	0	0	0	0	0	0

**a) 2010 vs 2013**

**Table:**

		**2010**									
		R11	R12	R13	R14	R15	R16	R17	R18	R19	R20
		(1)	(1)	(1)	(1)	(1)	(1)	(1)	(1)	(1)	(1)
	R1 (2)	0	0	0	0	0	0	0	0	0	0
	R2 (69)	0	0	0.01	0	0	0	0.01	0	0.01	0.01
	R3 (13)	0.03	0.38	0	0	0	0	0	0	0	0
**2013**	R4 (27)	0.02	0	0	0	0	0.03	0	0	0	0
	R5 (11)	0	0	0	0	0	0	0	0	0	0
	R6 (2)	0	0	0	0	0	0	0	0	0	0
	R7 (53)	0.03	0	0	0	0.01	0.01	0	0	0	0
	R8 (3)	0	0	0	0	0	0	0	0	0	0
	R9 (9)	0	0	0	0	0	0	0	0.11	0	0
	R10 (5)	0	0	0	0	0	0	0	0	0	0
	R11 (15)	0.45	0	0	0	0	0	0	0	0	0
	R12 (3)	0.06	0	0	0	0	0	0	0	0	0
	R13 (1)	0	0	0	1	0	0	0	0	0	0
	R14 (1)	0	0	0	0	0	0	0	0	0	0

**b) 2013 vs 2017 **

**Table:**

		**2013**								
		R1	R2	R3	R4	R5	R6	R7	R8	R9
		(5)	(29)	(59)	(16)	(13)	(82)	(7)	(2)	(1)
	R1 (59)	0	0	0.93	0	0	0	0	0.03	0
	R2 (96)	0	0	0.01	0	0.13	0.81	0	0	0
	R3 (6)	0.57	0	0	0	0	0.02	0	0	0
**2017**	R4 (16)	0	0	0	1	0	0	0	0	0
	R5 (28)	0	0.96	0	0	0	0	0	0	0
	R6 (7)	0	0	0	0	0	0	1	0	0
	R7 (1)	0.2	0	0	0	0	0	0	0	0
	R8 (1)	0	0	0	0	0	0	0	0	1

**Remittance vs Migration Communities **

**a) 2010 **

**Table:**

	R1	R2	R3	R4	R5	R6	R7	R8	R9	R10
	(67)	(3)	(74)	(7)	(4)	(15)	(26)	(5)	(1)	(1)
M1 (2)	0.01	0	0.01	0	0	0	0	0	0	0
M2 (9)	0	0	0.1	0	0	0	0.02	0	0	0
M3 (60)	0.58	0	0.05	0.01	0	0.01	0.02	0	0	0
M4 (22)	0.02	0	0	0	0	0.02	0.65	0	0	0
M5 (10)	0.01	0	0.12	0	0	0	0	0	0	0
M6 (5)	0.04	0	0.01	0	0	0	0	0	0	0
M7 (7)	0	0	0.05	0	0.37	0	0	0	0	0
M8 (61)	0.08	0	0.43	0	0.01	0.05	0.03	0	0	0.01
M9 (7)	0	0	0.01	0	0	0	0	0.71	0	0
M10 (2)	0	0.66	0	0	0	0	0	0	0	0
M11 (14)	0	0	0	0.4	0	0.38	0	0	0	0
M12 (5)	0.02	0	0	0	0	0.05	0.03	0	0	0
M13 (1)	0	0	0	0	0	0	0	0	1	0
M14 (1)	0	0.33	0	0	0	0	0	0	0	0
M15 (1)	0	0	0.01	0	0	0	0	0	0	0
M16 (1)	0	0	0	0	0	0	0	0	0	0
M17 (1)	0	0	0	0	0	0	0	0	0	0
M18 (1)	0.01	0	0	0	0	0	0	0	0	0
M19 (1)	0	0	0.01	0	0	0	0	0	0	0
M20 (1)	0	0	0	0	0	0	0	0	0	0
M21 (1)	0	0	0	0	0	0	0	0	0	0

**a) 2010 **

**Table:**

	R11	R12	R13	R14	R15	R16	R17	R18	R19	R20
	(1)	(1)	(1)	(1)	(1)	(1)	(1)	(1)	(1)	(1)
M1 (2)	0	0	0	0	0	0	0	0	0	0
M2 (9)	0	0	0	0	0	0	0	0	0	0
M3 (60)	0	0	0	0	0	0.01	0	0.01	0	0
M4 (22)	0	0	0	0	0	0	0	0	0	0
M5 (10)	0	0	0	0	0	0	0	0	0	0
M6 (5)	0	0	0	0	0.2	0	0	0	0	0
M7 (7)	0	0	0	0	0	0	0	0	0	0
M8 (61)	0	0	0.01	0	0	0	0	0	0	0
M9 (7)	0.14	0	0	0	0	0	0	0	0	0
M10 (2)	0	0	0	0	0	0	0	0	0	0
M11 (14)	0	0	0	0	0	0	0	0	0	0
M12 (5)	0	0.2	0	0	0	0	0	0	0	0
M13 (1)	0	0	0	0	0	0	0	0	0	0
M14 (1)	0	0	0	0	0	0	0	0	0	0
M15 (1)	0	0	0	0	0	0	0	0	0	0
M16 (1)	0	0	0	1	0	0	0	0	0	0
M17 (1)	0	0	0	0	0	0	1	0	0	0
M18 (1)	0	0	0	0	0	0	0	0	0	0
M19 (1)	0	0	0	0	0	0	0	0	0	0
M20 (1)	0	0	0	0	0	0	0	0	1	0
M21 (1)	0	0	0	0	0	0	0	0	0	1

**b) 2013 **

**Table:**

	R1	R2	R3	R4	R5	R6	R7	R8	R9
	(5)	(29)	(59)	(16)	(13)	(82)	(7)	(2)	(1)
M1 (2)	0	0	0	0	0	0	0	1	0
M2 (69)	0	0.2	0.64	0	0	0	0	0	0
M3 (13)	0.058	0.05	0.02	0	0.08	0.06	0	0	0
M4 (27)	0	0.8	0.02	0	0	0	0	0	0
M5 (11)	0	0	0.01	0	0.26	0.05	0	0	0
M6 (2)	0	0	0	0	0.15	0	0	0	0
M7 (53)	0	0	0	0.02	0	0.6	0	0	0
M8 (3)	0	0	0	0	0.23	0	0	0	0
M9 (9)	0	0	0	0	0	0.02	0.77	0	0
M10 (5)	0.66	0	0	0	0	0.01	0	0	0
M11 (15)	0	0	0.01	0.82	0	0	0	0	0
M12 (3)	0	0	0.05	0	0	0	0	0	0
M13 (1)	0	0	0	0	0.07	0	0	0	0
M14 (1)	0	0	0	0	0	0	0	0	1

**c) 2017 **

**Table:**

	R1	R2	R3	R4	R5	R6	R7	R8
	(59)	(96)	(6)	(16)	(28)	(7)	(1)	(1)
M1 (33)	0.08	0	0	0	0.69	0	0	0
M2 (13)	0	0.13	0	0	0	0	0	0
M3 (20)	0.01	0.1	0.3	0	0.02	0	0.05	0
M4 (53)	0.53	0.1	0	0	0	0	0	0
M5 (64)	0.07	0.48	0	0.01	0.02	0	0	0
M6 (2)	0.03	0	0	0	0	0	0	0
M7 (3)	0	0.03	0	0	0	0	0	0
M8 (16)	0.01	0	0	0.88	0	0	0	0
M9 (9)	0	0.1	0	0	0	0.77	0	0
M10 (1)	0	0	0	0	0	0	0	1

Jaccard Similarity Coefficients are used to assess the similarity of community membership. The methodology is outlined in Sect. 2. R and M represent remittance and migration communities, respectively, and the number of member economies is given in the parenthesis. A coefficient of 1 indicates that two communities have all members in common, and a coefficient of 0 indicates no members in common. Jaccard Indexes are used to compare year-on-year community formation for the Global Remittance Network and Global Migration Network, respectively. In both networks, the the integration of single-node communities into larger communities can be observed between 2010 and 2013. Communities stabilize after 2013, with coefficients of or near 1 between a 2013 community and its 2017 counterpart. Remittance and migration communities are also compared with each other for each year. By 2017, some medium-sized communities closely overlap, such as R4 with M16, and R6 with M9. The largest communities have close to half of their members in common, such as R1 with M4 or R2 with M5
